# Protocol for a qualitative study exploring older adults’ experience of mental health and wellbeing in work-related later life transitions in the UK

**DOI:** 10.1371/journal.pone.0318737

**Published:** 2026-03-02

**Authors:** Rebecca Woodhouse, Dean McMillan, Ruth Wadman

**Affiliations:** 1 Department of Health Sciences, University of York, York, United Kingdom; 2 Hull York Medical School, University of York, York, United Kingdom; UofM: The University of Memphis, UNITED STATES OF AMERICA

## Abstract

Later life transitions occur in the later years of life and can cause significant disruption to a person’s usual daily routine and lifestyle. Transitions that are specifically related to work, such as retirement, can cause sudden changes to an individual’s financial, social and psychological environment and for some, may negatively impact on mental health and wellbeing. This protocol outlines a qualitative study to explore the experiences of adults in the UK, who have experienced a negative impact on their mental health or wellbeing due to a work-related transition in later life. Semi-structed interviews will be completed with people aged 50 and over to gain a deeper understanding of 1) the mental health and wellbeing of people undertaking later life transitions; 2) the context surrounding the transitions (such as due to ill-health or redundancy); and 3) the types of support they received or may have benefitted from. Research in this area is scarce and this study will help us to better understand the experience of those who have encountered such difficulties to help develop appropriate interventions and support in this area.

## Introduction

Later life transitions are periods of change that occur in the later years of life that have a significant impact on a person’s usual lifestyle or environment. As these types of transitions create such significant changes, including psychological, social and economic, they can sometimes have a negative impact on mental health and wellbeing [1,2]. Later life transitions involve any significant period of change but often LLTs refer to bereavement, relocation to a new housing type, such as a care home, and work-related transitions such as retirement. This study is specifically focussed on transitions that are related to work, including, role or career change, a reduction in hours from full to part time, moving to a type of ‘bridge-employment’ (paid work undertaken after main career job, but before full retirement), retirement and volunteering.

Retirement is the transition that tends to be most frequently reported on. It can force a wave of changes on an individual, that may be sudden and somewhat unexpected, impacting both mentally and physically [3]. Transitioning from employment to retirement often leads to the removal or reduction in financial security, fewer opportunities for socialisation, losing a sense of accomplishment and a regular routine; often factors in keeping well [1]. Retirement and other work-related transitions are experienced differently for everyone and the contexts that surround them are also widely varied [3,4]. Contexts surrounding these transitions and the adjustment that follows can include time to prepare, personal choice, and social circumstances [5].

There are findings reporting both positive and negative experiences for later life transitions, specifically for retirement. A recent review reported a third of retirees had depression during their retirement transition [6], whist another has shown retirement can be beneficial by improving depressive symptoms [7] or reducing the risk of depression by nearly 20% [8]. Various factors may be able to account for some of the variations, for example, those forced to undertake a transition, such as involuntary retirement due to redundancy or ill-health, led to poorer health outcomes compared to those not forced to retire [9]. Other contextual variations may also be of importance impacting on a person’s direct experience of undertaking a LLT. These include, a person’s socio-economic status, whereby those with a higher SES may be more likely to experience improved mental health compared with those with lower SES [10], health status determining the underlying reason for the transition and gender differences [11], Though, the research surrounding this is primarily on the retirement transition and often the contexts are multifaceted. In addition, the majority of existing studies are not focussed on the transition period itself with inconsistent time periods since retirement reported, with some exploring wellbeing in retirement with participants over 10 years since the actual transition.

Existing research has identified a number of factors that have impacted on a person’s daily life and wellbeing during retirement. One that is often reported is the changes experienced to person’s identity and role, which have a strong impact on a person’s daily life once they are retired [12,13]. Social interactions and relationships were of also noted to be of value, with an important need for emotional adjustment following retirement [12–18]. These findings are important, to highlight the particular values and factors that are disrupted during later life transitions; though the studies are rather varied in their reporting periods (time since the transition) and are predominantly samples of retirees within general populations, not specifically those who have experienced a negative impact on mental health or wellbeing.

The lived experience of people undertaking work-related transitions in later life has been sparsely reported, especially for non-retirement work-related transitions such as late life career or role change. For retirement, existing qualitative studies have focussed on general populations of retirees rather than those who have specifically experienced a negative impact on their mental health or psychological wellbeing due to the transition. There is little (if any) published research focussing on the direct experiences of those who have experienced a negative impact on their mental health or psychological wellbeing in work-related later life transitions.

As research has reported, some people may struggle with their mental health and psychological wellbeing during or following a work-related transition in later life. It is therefore important to understand the experiences, both positive and negative, of those who have encountered such difficulties to develop appropriate interventions and support in this area.

## Materials and methods

Aims: This study aims to explore the direct experiences of people who have experienced a negative impact on their mental health or psychological wellbeing due to undertaking a work-related transition in later life. The main areas of focus will be the experience and context of the transition and exploring the impact of the transition on mental health and wellbeing. The support and resources used by individuals will also be explored to identify factors that may have helped or hindered the transition period. In addition, the interviews will gather people’s perspectives on what help or support would have been acceptable to them during the transition: when the most beneficial timing to receive support would be; what that support may look like or involve; and who might provide it. This information will help to shape the future development of interventions to support people through work-related later life transitions.

Research objectives:

1. To explore the views and experiences of people who have experienced a negative impact on their mental health or wellbeing due to undertaking a work-related transition in later life.
2. To explore the support and resource use of people undertaking work-related later life transitions.
3. To identify acceptable supports and resources for work-related later life transitions to inform future research and the development of suitable interventions to support these transitions.

### Study design

This is an exploratory qualitative study using individual semi-structured interviews to explore the positive and negative experiences and views of older adults who have recently undertaken a work-related transition. This type of interview was deemed to be the most appropriate due to the potentially wide variations in transitions, employment type and role, which may make group discussions difficult due to the uniqueness of everyone’s transition context. In addition, due to the sensitive nature of the discussions on mental health and wellbeing, focus groups were not chosen as they can limit people’s willingness to be open about their personal experiences without feeling embarrassed or feeling judgement, potentially limiting the depth of the results [19]. Though focus groups may have been fruitful in encouraging some discussion and offering different perspectives, they are particularly useful when studying a particular culture or group [19]. Individual interviews can be easily conducted remotely, via telephone or video call, making it possible to include participants across a wide geographical region.

### Participants

Participants will be aged 50 and over and will have undertaken a work-related transition in the past five years, which has negatively impacted on their mental health or wellbeing. The transition will be related to work or employment, such as retirement, role or career change, or move to part time work or volunteering. The mental health and wellbeing difficulties will be self-identified by participants and not assessed using a standardised diagnostic measure.

### Inclusion criteria

- Adults aged 50 and over
- Undertaken a work-related transition in the past five years
- Experienced negative impact on their mental health or wellbeing around the same period as the LLT (self-reported).
- Living in the UK

### Exclusion criteria

- Unable to read and speak English

### Sample size

A minimum of ten and maximum of 30 participants will be sought, though this will remain flexible. Interviews (above ten) will continue until a sufficient depth of understanding is achieved relating to the research objectives [20].

A varied sample will be sought, including a mixture of males and females, a range of ages (50 and over), and different occupational contexts (including type of workplace and roles held). Retirement is predicted to be the most reported transition, but recruitment will be open to other work-related transitions (such as role or career change).

### Recruitment

Purposive sampling will be utilised to ensure a broad sample is recruited, representing a range of transitions, participant characteristics and contexts. As this research aims to explore a range of work-related transitions, rather than just focussing on retirement, this sampling strategy is appropriate to ensure different perspectives, contexts and experiences are included [21]. A simple matrix will be created to ensure key groups are represented and a diverse selection is recruited (including sex, transition type etc). This sampling method is consistent with reflective thematic analysis, where the aim of recruitment is to generate purposeful, rich, in-depth accounts of lived experiences relevant to the research topic within a particular context, rather than to achieve statistical generalisability [22]. Basic demographic information will be obtained for recruitment and contact purposes and to ensure a range of LLTs and LLT contexts (planned/unplanned) are represented.

Various recruitment techniques will be utilised to ensure a wide range of participants are identified. The study will be advertised via poster, leaflet or email in a selection of relevant ‘older adult’ locations, including community centres, retirement groups, mailing lists such as the University of York Pension groups, local cafes and businesses and communal spaces and on social media.

If required, for recruitment purposes, study information packs containing an invite letter, participant information sheet, and example consent form, may also be available in some of the relevant locations (described above).

Recruitment and data collection for this study has begun and both are expected to be completed by the end of May 2025.

### Consent process

Potential participants will be invited to contact the lead researcher (RW) by telephone or email to express their interest in taking part. The researcher will explain what the study involves and if the potential participant is interested, the researcher will ask their preference for receiving the study information pack via post or email. The study information pack includes a consent form, participant details form, participant information sheet and invite letter, along with a stamped addressed envelope (if postal). The participant information sheet describes the study purpose and processes involved, including what would be involved for the participant, the withdrawal procedure, confidentiality and data management. Participants are required to consent to being audio-recorded in order to take part in the study.

There will be several options for obtaining informed consent from participants: 1) the participant returns a competed and signed consent form in the post, 2) the participant returns a completed and signed consent form via email, or 3) the participant provides audio-recorded verbal consent prior to their qualitative interview. Once the consent form (and participant details form) is returned (via post or email), the researcher will make contact via phone or email (participant preference) to arrange the interview.

Participants will also complete a participant details form. These details are collected for contact and sampling purposes.

### Procedure (data collection)

The researcher will arrange a day and time to conduct the interview, arranged to be most convenient for the participant. Semi-structured individual interviews will take place over the telephone or using a remote online platform such as Zoom (dependant on participant preference) and will be audio-recorded. A predefined topic guide will be used to guide the interviews and includes questions and prompts relating to the context of the transition (including voluntary/forced), the impact on mental health and wellbeing and positive experiences from the transition. The topic guide has been reviewed by PPI advisory group members with experience of undertaking a later-life transition and refined accordingly. A mock interview was also held with a PPI member to test the topic guide, consent and audio-recording processes. Initially the topic guide will be piloted with two participants and will be refined as needed for subsequent interviews. Specifically, the pilot interviews will be used to assess the relevance and clarity of the questions, ensure the questions are eliciting detailed responses from participants and help to identify areas that may require greater depth or flexibility, including the associated question prompts. Interviews will be conducted by the lead researcher (RW) and will last approximately 30–45 minutes.

Example questions from the topic guide:

1. *What was your experience of the transition?*

PROMPT – how did you feel prior to [*retirement/other*], how did you find the transition period? Did you feel well supported (employees/family)

1. *Can you describe any difficulties you experienced with your mental health and wellbeing?*
2. *What, if anything, do you think would have helped you during this time?*

### Analysis

The audio-recordings from the interviews will be transcribed verbatim by the lead researcher (RW). The transcripts will be initially read by the lead researcher, to allow the researcher to become familiar with the data and will then be descriptively coded. Following guidelines from Braun and Clarke [23], a second researcher will not be sought (to code a selection of the transcripts) to test validity of the coding. Within reflective thematic analysis, data meanings are subjective, and codings are open to different interpretations. Attempting to use a second researcher to code transcripts to assess inter-rater reliability is therefore considered to be redundant as reflective thematic analysis is flexible and will vary across researchers. An appropriate coding software such as NVivo will be used.

The qualitative method of reflective thematic analysis, of those directly involved in LLTs will be used to inductively identify important or interesting patterns and themes within the data [24]. The framework put forward by Braun and Clarke [24], describes a six-step process for conducting thematic analysis. This process will be followed for the analysis of the study data. Thematic analysis is an appropriate method of analysis to understand experiences, thoughts, and behaviours within a set of interview data [25].

The six steps of thematic analysis are

Step one:

Become familiar with the data – by reading and re-reading the transcripts, relistening to audio recordings and making reflective notes.

Step two:

Generate initial codes – reflective and iterative meaningful organisation of the data considering the study aims and research questions; with a focus on what is being said rather than attempting a deeper interpretation. The process is interpretive and shaped by the researcher’s engagement with the data. Transcripts may be revisited, as required, as new insights emerge.

Step three:

Search for themes – identifying interesting or significant patterns of shared meaning within the data to produce preliminary themes. This step involves moving from codes to broader patterns. These patterns contain interesting or meaningful insights from the data in relation to the research question, to attempt to explain the experiences.

Step four:

Review themes – modify and develop the themes identified in step three, considering their level of coherency and uniqueness from each other. This involves ensuring the themes are distinctive and meaningful and represent the patterns within the data. The process is iterative and earlier steps may be revisited as required. Consistent with reflective thematic analysis, the process does not involve any formal validation of themes, rather transparent, reflective sense-making and interpretation by the researcher.

Step five

Define themes – confirming the themes and subthemes, how they interact and the meaning behind them. This involves articulating what is significant about each theme and how each relates to each other and the research questions.

Step six:

The write-up – drafting of the full report to detail the study processes and findings, ensuring the researcher’s role is acknowledged throughout.

The analysis and write up for this study are expected to be completed by September 2025.

### Data management

Participant identifiable materials (i.e., consent forms, participant details forms) will be stored on secure password-protected computers or in locked filing cabinets at the University of York and will only be accessible by the research team. Audio-recordings will be given a unique code and will be uploaded onto a secure password-protected computer. These recordings will be anonymised during the transcription process with any identifiable information removed. Audio-recordings will be deleted from the voice recorder once the transcription process is complete. Signed consent forms, audio-recordings and transcripts will be securely stored for up to 10 years after the study has completed, after which will be deleted or destroyed.

### Ethics and risks

Ethical approval for this study was obtained from the University of York, Department of Health Sciences Research Governance Committee on 19^th^ May 2023 (ref. HSRGC/2023/566/B). The study data will be confidential and anonymised throughout the study period and dissemination. Participants will be allocated a unique ID for the study. The study is currently recruiting, and it is anticipated the study will be completed and written up by the end of September 2025.

### Dissemination

This study will be written up as part of a PhD thesis and will be published in a relevant academic journal and may be presented at research conferences. All participants involved in the study will be offered a summary of the findings. This will be sent via email with a postal option for participants where email is not a feasible option.

## Discussion

This qualitative study will add to the limited literature in this area, to provide an in-depth insight into how work-related later life transitions are experienced by people who have experienced a negative impact on their mental health or wellbeing due to the transition. The study will help to inform future research looking to develop supports for people undertaking such transitions, by providing a richer understanding of the context, experience and support surrounding the different transitions.

One potential risk to this study is ensuring a diverse sample covering the different types of work-related transitions, the context of the transition (voluntary vs unvoluntary) and the different types of job roles. To mitigate this risk, the study will employ a range of recruitment methods to target potential participants across different geographical locations, organisations, and groups. The recruitment strategy will be monitored and refined if required.

In summary, the study will explore the direct experience of those who have recently undertaken a work-related later life transition, in relation to their mental health and wellbeing. This will help inform future research to develop suitable forms of support of people undertaking such transitions.

## References

[1] Dang L, Ananthasubramaniam A, Mezuk B. Spotlight on the Challenges of Depression following Retirement and Opportunities for Interventions. Clin Interv Aging. 2022;17:1037–56. doi: 10.2147/CIA.S336301 35855744 PMC9288177

[2] Pachana NA, Laidlaw K, Munk KP. Transitions in Later Life. The Oxford Handbook of Clinical Geropsychology. Oxford University Press. 2014. doi: 10.1093/oxfordhb/9780199663170.013.006

[3] Dave D, Rashad I, Spasojevic J. The effects of retirement on physical and mental health outcomes. Southern Economic Journal. 2008;75(2):497–523.

[4] Scharn M, Sewdas R, Boot CRL, Huisman M, Lindeboom M, van der Beek AJ. Domains and determinants of retirement timing: A systematic review of longitudinal studies. BMC Public Health. 2018;18(1):1083. doi: 10.1186/s12889-018-5983-7 30170592 PMC6119306

[5] Liu H, Fang B, Chan J, Lou VW. Continued social participation protects against depressive symptoms across the retirement transition: Longitudinal evidence from three waves of the China Health and Retirement Longitudinal Survey. Geriatr Gerontol Int. 2019;19(10):972–6. doi: 10.1111/ggi.13752 31397048

[6] Pabón-Carrasco M, Ramirez-Baena L, López Sánchez R, Rodríguez-Gallego I, Suleiman-Martos N, Gómez-Urquiza JL. Prevalence of Depression in Retirees: A Meta-Analysis. Healthcare (Basel). 2020;8(3):321. doi: 10.3390/healthcare8030321 32899813 PMC7551681

[7] van der Heide I, van Rijn RM, Robroek SJW, Burdorf A, Proper KI. Is retirement good for your health? A systematic review of longitudinal studies. BMC Public Health. 2013;13:1180. doi: 10.1186/1471-2458-13-1180 24330730 PMC4029767

[8] Odone A, Gianfredi V, Vigezzi GP, Amerio A, Ardito C, d’Errico A, et al. Does retirement trigger depressive symptoms? A systematic review and meta-analysis. Epidemiol Psychiatr Sci. 2021;30:e77. doi: 10.1017/S2045796021000627 35048820 PMC8679838

[9] Donahue P. Retirement reconceptualized: forced retirement and its relationship to health. Canada: University of Toronto. 2006.

[10] Vigezzi GP, Barbati C, Maggioni E, Stenholm S, Odone A, Italian Working Group on Retirement and Health, et al. Impact of retirement transition on health, well-being and health behaviours: critical insights from an overview of reviews. Soc Sci Med. 2025;375:118049. doi: 10.1016/j.socscimed.2025.118049 40250262

[11] Jokela M, Ferrie JE, Gimeno D, Chandola T, Shipley MJ, Head J, et al. From midlife to early old age: health trajectories associated with retirement. Epidemiology. 2010;21(3):284–90. doi: 10.1097/EDE.0b013e3181d61f53 20220519 PMC3204317

[12] Cahill M, Galvin R, Pettigrew J. The retirement experiences of women academics: a qualitative, descriptive study. Educational Gerontology. 2021;47(7):297–311. doi: 10.1080/03601277.2021.1929266

[13] Hobbis S, Thirlaway K, Sanders L, Hendry L. Research: Retirement and lifestyle behaviours: A thematic analysis of a pilot study. bpshpu. 2011;20(2):2–8. doi: 10.53841/bpshpu.2011.20.2.2

[14] Bauger L, Bongaardt R. The lived experience of well-being in retirement: A phenomenological study. Int J Qual Stud Health Well-being. 2016;11:33110. doi: 10.3402/qhw.v11.33110 27814778 PMC5097159

[15] Fadeeva A. Promoting health and well-being in later life: retirement as a transition point. Northumbria University. 2020.

[16] Genoe MR, Liechty T, Marston HR. Leisure innovation and the transition to retirement. Leisure Sciences. 2019;44(4):440–58. doi: 10.1080/01490400.2019.1597791

[17] Haigh J. Women’s retirement: The self in process of transition. Sheffield Hallam University. 2005.

[18] Pepin G, Deutscher B. The Lived Experience of Australian Retirees: ‘I’m Retired, What Do I Do Now?’. British Journal of Occupational Therapy. 2011;74(9):419–26. doi: 10.4276/030802211x13153015305556

[19] Kitzinger J. Qualitative research. Introducing focus groups. BMJ. 1995;311(7000):299–302. doi: 10.1136/bmj.311.7000.299 7633241 PMC2550365

[20] Braun V, Clarke V. To saturate or not to saturate? Questioning data saturation as a useful concept for thematic analysis and sample-size rationales. Qualitative Research in Sport, Exercise and Health. 2019;13(2):201–16. doi: 10.1080/2159676x.2019.1704846

[21] Robinson OC. Sampling in Interview-Based Qualitative Research: A Theoretical and Practical Guide. Qualitative Research in Psychology. 2013;11(1):25–41. doi: 10.1080/14780887.2013.801543

[22] Braun V, Clarke V. Reflecting on reflexive thematic analysis. Qualitative Research in Sport, Exercise and Health. 2019;11(4):589–97. doi: 10.1080/2159676x.2019.1628806

[23] Braun V, Clarke V. Conceptual and design thinking for thematic analysis. Qualitative Psychology. 2022;9(1):3–26. doi: 10.1037/qup0000196

[24] Braun V, Clarke V. Using thematic analysis in psychology. Qualitative Research in Psychology. 2006;3(2):77–101. doi: 10.1191/1478088706qp063oa

[25] Braun V, Clarke V. Thematic analysis. APA handbook of research methods in psychology. Washington, DC, US: American Psychological Association. 2012. 57–71.

